# Diminished but Not Abolished Effect of Two His351 Mutants of Anthrax Edema Factor in a Murine Model

**DOI:** 10.3390/toxins8020035

**Published:** 2016-02-02

**Authors:** Taoran Zhao, Xinghui Zhao, Ju Liu, Yingying Meng, Yingying Feng, Ting Fang, Jinlong Zhang, Xiuxu Yang, Jianmin Li, Junjie Xu, Wei Chen

**Affiliations:** 1 Laboratory of Vaccine and Antibody Engineering, Beijing Institute of Biotechnology, Beijing 100071, China; tracyztr@163.com (T.Z.); 18701522421@163.com (J.L.); myy316@163.com (Y.M.); fangting1008vip@163.com (T.F.); zjl0679@163.com (J.Z.); yangxiuxu118@163.com (X.Y.); lijmqz@126.com (J.L.); 2 Department of Colorectal Surgery, the Second Artillery General Hospital, Beijing 100088, China; fyy19811120@163.com

**Keywords:** anthrax toxin, edema factor, evaluation model

## Abstract

Edema toxin (ET), which is composed of a potent adenylate cyclase (AC), edema factor (EF), and protective antigen (PA), is one of the major toxicity factors of *Bacillus anthracis*. In this study, we introduced mutations in full-length EF to generate alanine EF(H351A) and arginine EF(H351R) variants. In vitro activity analysis displayed that the adenylyl cyclase activity of both the mutants was significantly diminished compared with the wild-type EF. When the native and mutant toxins were administered subcutaneously in a mouse footpad edema model, severe acute swelling was evoked by wild-type ET, while the symptoms induced by mutant toxins were very minor. Systemic administration of these EF variants caused non-lethal hepatotoxicity. In addition, EF(H351R) exhibited slightly higher activity in causing more severe edema than EF(H351A). Our findings demonstrate that the toxicity of ET is not abolished by substitution of EF residue His351 by alanine or arginine. These results also indicate the potential of the mouse footpad edema model as a sensitive method for evaluating both ET toxicity and the efficacy of candidate therapeutic agents.

## 1. Introduction

*Bacillus anthracis* is a highly pathogenic bacterium that is the causative agent of anthrax, an acute infectious disease that is lethal to both humans and animals. The virulence factors of this pathogen contain two main aspects: the anti-phagocytic poly-d-glutamic acid capsule [1] and anthrax toxins. The three toxin components produced by *B. anthracis* are protective antigen (PA), lethal factor (LF), and edema factor (EF). The two catalytic components, LF and EF, are delivered into the cytosol via receptor binding of component PA in the form of the lethal toxin (LT) complex composed of LF and PA, and the edema toxin (ET) complex composed of EF and PA [2]. LF is a zinc-dependent metalloproteinase which cleaves the *N*-terminus of mitogen-activated protein kinase kinases (MAPKK) 1–4, 6, and 7 [3], while EF is a potent calmodulin-dependent adenylate cyclase (AC) which modulates cell signaling pathways by increasing intracellular cAMP levels. EF is an 89 kDa soluble protein, which contributes to pathogen distribution, tissue damage, and lethality. Systemic administration of purified EF to mice leads to intestinal intraluminal fluid accumulation, hemorrhaging in the ileum and adrenal glands, a variety tissue lesions, and the induction of several inflammatory factors [4].

However, EF is inactive outside the host cell. Calmodulin (CaM), an endogenous Ca^2+^ ion sensor, is naturally present in host cells. Upon interaction with CaM, EF undergoes marked conformational changes, ultimately resulting in high catalytic AC activity. EF consists of three modular domains, a 30 kDa *N*-terminal PA-binding domain, a 43 kDa AC domain, and a 17 kDa helical domain [5]. The AC domain can be divided structurally into the CA and CB domains, with the catalytic site located at the interface. The EF helical domain binds to the *N*-terminal domain of CaM, allowing insertion of the *C*-terminal between the catalytic core and helical domains of EF. This initiates a conformational change, in which the critical catalytic loop of EF with high AC activity (approximately 1000–2000 molecules per second) is stabilized through the switch C region [6]. Various residues that are directly related to substrate binding have been identified, including a histidine at position 351 (H351) [5,7]. H351 is located in the catalytic cleft of EF and interacts with the reactive 3′-hydroxyl group of ATP, acting as a catalytic base in the cyclization process. The substitution of H351 by alanine (A) or arginine (R) leads to a reduction in catalytic activity without altering the half-maximal effective concentration (EC_50_) values for CaM [8].

Despite decades of research, anthrax continues to be a threat to both humans and animals. While vaccines and antibiotics are available, the current antibiotic treatments are not suitable for all populations, thus, highlighting the need for novel strategies to treat anthrax infection as well as anti-toxin therapies that can be used in the case of toxin accumulation in the bloodstream [9]. EF is a highly efficient AC, regulating biological processes through a protein kinase A pathway, although this is not the only mechanism. EF was found to deplete cellular ATP, upregulate toxin receptors [10], and bind to other nucleotide triphosphates as substrates [11]. The existence of these other mechanisms underlying EF function demonstrates that candidate therapeutic agents cannot be identified on the basis of AC repression alone. Furthermore, EF causes cellular or tissue damage through regulating a variety of inflammatory factors. Thus, a comprehensive evaluation of all the possible effects on EF function is likely to be time-consuming and highly expensive; therefore, accurate and convenient methods for evaluation of the inhibition or neutralization of EF toxicity are required. Subcutaneous injection of ET into mice causes swelling, which can be abolished by the administration of a monoclonal anti-EF antibody [12]. In this study, H351 mutants (H351A and H351R), which diminished EF AC activity were expressed and purified. The ability of these mutants to decrease the AC activity of EF was evaluated in a mouse model of footpad edema induced by subcutaneous administration of ET. Observation of the pathogenesis of edema indicated the potential of this model for the assessment of therapeutic candidates.

## 2. Results and Discussion

### 2.1. Site-Directed Mutagenesis, Expression, and Purification of Mutant EF Proteins

**Figure 1:**
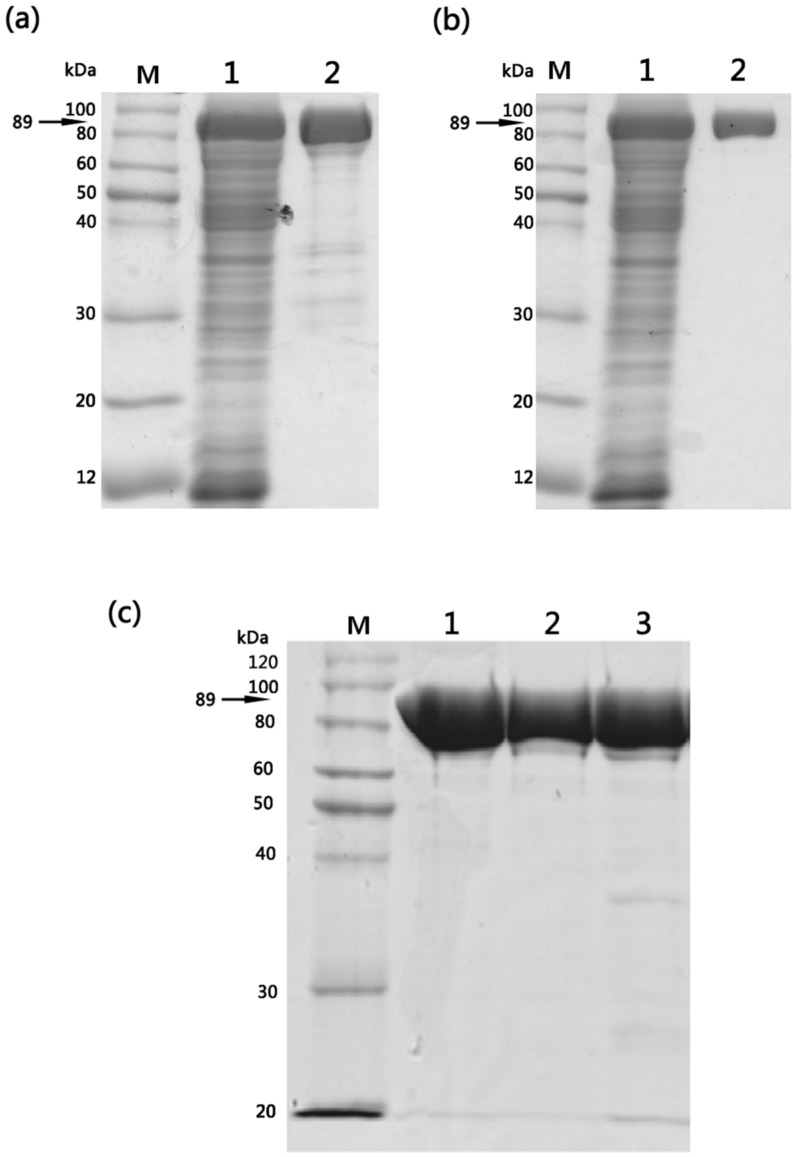
Purification of wild-type EF, EF(H351A) and EF(H351R) proteins expressed in *E. coli* M15 strain. (**a**) SDS-PAGE analysis of Ni-NTA affinity purified wild-type EF: M, molecular weight marker; lane 1, total soluble fraction; lane 2, elution fraction; (**b**) SDS-PAGE analysis of SP-sepharose cation-exchange chromatography purified wild-type EF: M, molecular weight marker; lane 1, total soluble fraction; lane 2, elution fraction. Similar results were obtained following purification of EF(H351A) and EF(H351R) proteins using the same procedures; (**c**) SDS-PAGE (10% gel) analysis of 1 μg of purified EF, EF(H351A), and EF(H351R): M, molecular weight marker; Lane 1, EF; lane 2, EF(H351A); lane 3, EF(H351R).

The pQE30-EF(H351A) and pQE30-EF(H351R) expression plasmids containing mutations of His351 to alanine (A) or arginine (R), respectively, within the catalytic site of EF [8] were confirmed by sequencing. According to previous reports, circular dichroism profiles indicate that the mutant proteins do not exhibit any gross structural differences compared to the wild-type EF [8] and remain competent in binding to PA [13]. The wild-type and mutant proteins were expressed as *N*-terminal (His)_6_-tagged fusion proteins in *E. coli* M15 strain. Following induction with isopropyl-1-thio-β-d-galactopyranoside (IPTG), the cells were harvested and the periplasmic fraction was isolated. After Ni-NTA affinity chromatography and SP-sepharose cation-exchange column, wild-type EF or mutant proteins were isolated from other *E. coli* cell constituents. Purified wild-type and mutant proteins displayed identical purification characteristics (>90%) with respect to their molecular mass (89 kDa) and final yields (5–6 mg/L of culture) and sodium dodecyl sulfate-polyacrylamide gel electrophoresis (SDS–PAGE) analysis confirmed that homogenous preparations of structurally stable mutants were obtained (Figure 1).

### 2.2. Adenylate Cyclase Activity of Wild-Type and Mutant Proteins

**Figure 2:**
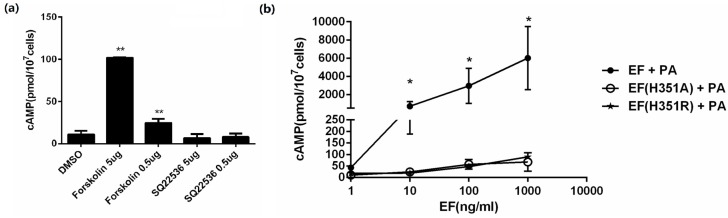
In vitro adenylate cyclase activity assay of EF, EF(H351A), or EF(H351R). (**a**) CHO cells were incubated for 2 h with forskolin (50 μg/mL and 5 μg/mL), SQ22536(50 μg/mL and 5 μg/mL) or DMSO control. Intracellular cAMP was measured using an EIA kit; (**b**) CHO cells were incubated for 2 h with PA (1 μg/mL) and EF, EF(H351A), or EF(H351R) at the indicated concentrations. Intracellular cAMP was measured using an EIA kit. An average of 13 pmol of cAMP/10^7^ CHO cells was detected in control cells (untreated cells or cells treated with only one toxin component). Results are expressed as mean ± standard error. Statistical significance: (** *p* < 0.01, * *p* < 0.05).

EF is a calmodulin-dependent AC, which elevates intracellular cAMP levels in the presence of PA. The cAMP level in CHO cells was measured within 2 h exposed to the expressed proteins accompanied with PA, and to forskolin or SQ22536. As expected, forskolin, an AC activator, could improve the intracellular cAMP level, while SQ22536, an AC inhibitor, decreased the cAMP level (Figure 2a). To verify the activity of the expressed proteins, CHO cells were treated with varying concentrations (0.5–500 ng/mL) of EF, EF(H351R), and EF(H351A) in the presence of 1 μg/mL PA for 2 h. A sharp increase in cAMP was observed in a dose-dependent manner when CHO cells were exposed to EF combined with PA, while a relatively mild but not significant increase when exposed to EF(H351R) or EF(H351A) (Figure 2b). Non-cellular reaction systems containing MgCl_2_, calmodulin, CaCl_2_, BSA, and ATP are commonly used to analyze the activity of EF or its mutants, but the detection sensitivity can be variable due to the difference in formulas. Previous evaluation of the EF(H351A) and EF(H351R) mutants in an in vitro AC assay showed a 200-fold reduction in the catalytic activity of these two mutant proteins compared to that of the native EF [5]. However, in another study, the AC activity of EF(H351A) was below the detectable limits of a similar, but not identical, analysis system [7]. So whether the substitution of His351 by alanine or arginine of EF remains AC activity of physiological and pathological impact may need further evaluation.

### 2.3. Footpad Edema Induced by Wild-Type and Mutant Toxins

Although ET is an important toxin relevant to anthrax pathogenesis, it plays a more comprehensive role in the aspects of bacterial infection that are not related to lethality. ET causes edema through targeting of an unknown cellular target (not endothelial cells) in the early stages of infection. Subcutaneous injection of ET induces edema in experimental animals [8,14] and a mouse model of footpad edema induction has been used to evaluate the effect of antibodies against ET [12]; therefore, we used this model to further investigate the activity of EF(H351A) and EF(H351R).

**Figure 3:**
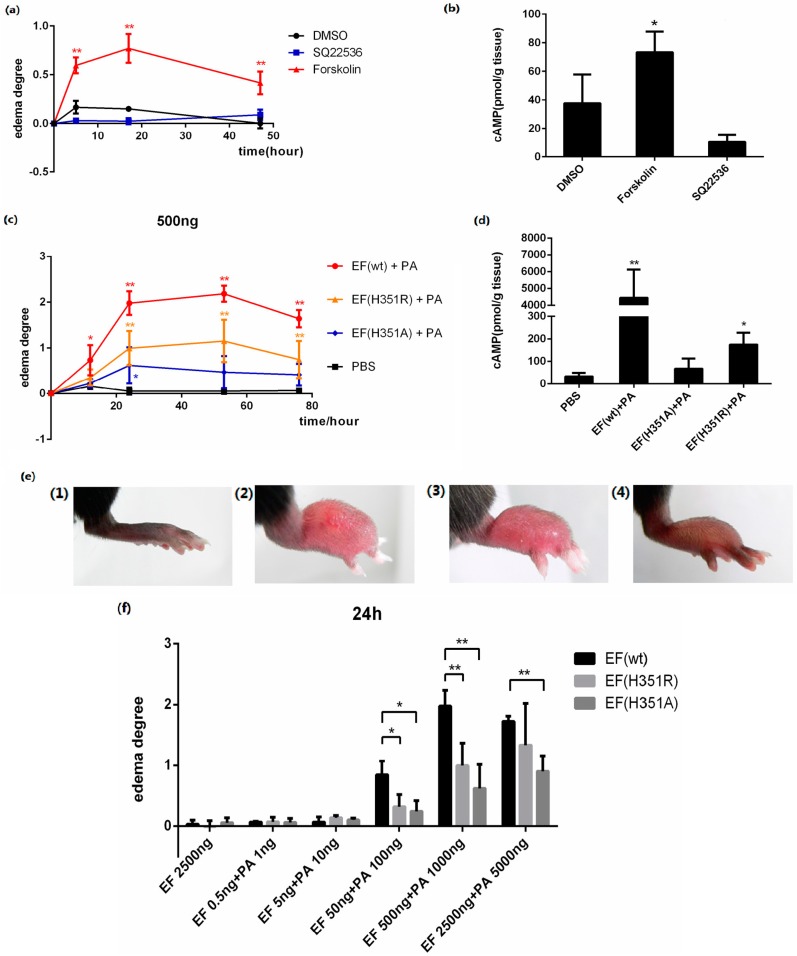

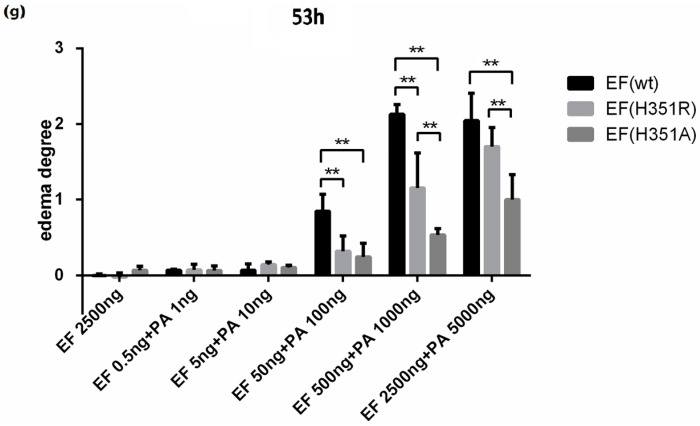
ET-induced footpad edema in mice. 25 μg of forskolin or SQ22536 dissolved in 25 μL DMSO were injected subcutaneously into the footpad; 25 μL of DMSO was administered as the solvent control. (**a**) Footpad thickness was measured at 0, 5, 17, and 49 h after injection; (**b**) cAMP levels in the treated footpads tissue. Mice were injected subcutaneously with 500 ng EF and 1000 ng PA (in 25 μL PBS), 500 ng EF(H351R) and 1000 ng PA or 500 ng EF(H351A) and 1000 ng PA; (**c**) Footpad thickness was measured at 0, 12, 24, 53, and 76 h after injection; (**d**) cAMP levels in the treated footpads tissue; (**e**) Comparison of right footpads treated with 1000 ng PA only (1), 500 ng EF and 1000 ng PA (2), 500 ng EF(H351R) and 1000 ng PA (3), or 500 ng EF(H351A) and 1000 ng PA (4). Mutant EF(H351A) induced modest edema, while wild-type EF caused much higher levels of edema; (**f**) and (**g**) Serial doses (0.5, 5, 50, 500, and 2500 ng) of EF, EF(H351R) or EF(H351A) in combination with double concentrations of PA were subcutaneously injected into the footpad; the degree edema at 24 h and 53 h after injection are shown. Significant differences between the groups are indicated by *p*-values. The *p*-values of the indicated groups versus the solvent control group are shown. Data represent the mean ± standard error of the mean based on *n* = 3 mice per treatment. At least two biological replicates were performed for each experiment. (** *p* < 0.01, * *p* < 0.05).

Forskolin is an AC activator which elevates the intracellular cAMP level, and SQ22536 is an AC inhibitor which decreases the intracellular cAMP level. In this study, 25 μg of each activator or inhibitor was injected subcutaneously into the footpads of mice. Local edema was induced by forskolin with an increase of cAMP in the tissue, but not by SQ22536 (Figure 3a,b), demonstrating that edema was induced by the elevation of intracellular cAMP.

The progression of lesions induced in the mouse footpad by subcutaneous ET injection was then observed. When 500 ng EF was administered in combination with 1000 ng PA, rapid footpad swelling was observed in experimental animals (Figure 3c), with peak swelling (footpad thickness) and ulceration detected after approximately 53 h passed (Figure 3e). Modest edema was observed following application of identical amounts of mutant toxins, EF(H351A) or EF(H351R) in combination with PA (Figure 3c–e). Meanwhile, the cAMP levels in footpads were increased about 100 times by native ET, four times by ET (H351R), and two times by ET (H351A) in 2 h of treatment (Figure 3d). No edema was observed following administration of EF, EF(H351A), EF(H351R), or PA (data not shown). The lesion caused by native or mutant ET challenge gradually healed within two weeks without any intervention (data not shown). The edema induction effects of varying amounts of wild-type or mutant toxins were also studied, and the peak edema values observed at 24 h and 53 h after injection were analyzed (Figure 3f,g). Although neither the wild-type nor mutant EFs induced edema at very low doses (0.5 ng and 5 ng), toxic effects emerged when the challenge dose was increased to 50 ng. Significant differences (*p* < 0.05) observed in the degree of edema caused by wild-type EF and the mutants, while there were no significant differences in the effects caused by EF(H351R) and EF(H351A). When the toxin challenge doses were elevated from 500 ng to 2500 ng, the degree of edema caused by the wild-type EF did not increase, while that caused by EF(H351R) increased such there was no significant difference between the effects of the wild-type EF and EF(H351R) (Figure 3d,e). These results indicate that the sensitivity of this mouse footpad edema model is sufficient to distinguish not only the weak toxicity of EF(H351R) and EF(H351A), but also increased toxicity of EF(H351R) compared with EF(H351A).

### 2.4. Systemic Effects of Wild-type and His351 EF Mutants

Systemic administration of ET *in vivo* results in a wide variety of organ lesions caused by the AC activity of EF, while PA alone is not toxic [15]. Subcutaneous injection of ET into the mouse footpad causes dramatic local edema, while systemic administration induces liver edema, fluid accumulation in the intestinal lumen, diverse tissue damage, and death with a minimum lethal dose for mice between 20 and 30 μg [14]. EF(H351A) is considered to be a potential prophylactic agent for anthrax based on its lack of toxicity in sensitive cell lines and its non-lethal effects when administered to mice in combination with an equal dose of PA [13]. However, the present study indicates that His531 mutation does not result in complete abolition of the toxicity of EF and therefore, caution should be exercised in using such mutants to prevent anthrax infection.

**Figure 4:**
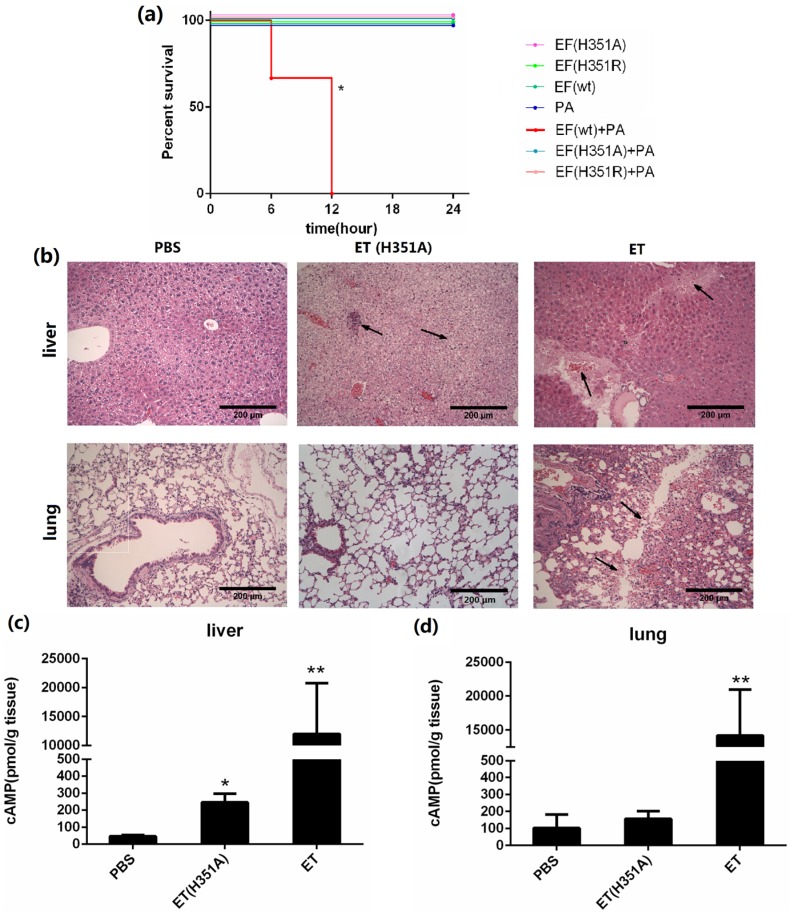
Systemic effects of wild-type and His351 EF mutants. (**a**) Survival rate of groups of three C57BL/6 mice challenged with wild-type or mutant EF (50 μg) and PA (100 μg). Mice challenged with EF (wild-type) + PA died within 12 h, while none died in the groups challenged with EF(H351R) + PA or EF(H351A) + PA, or in the control groups (EF or PA only). Mice were injected intravenously with PBS, EF (100 μg) and PA (200 μg), or EF(H351A) (100 μg) and PA (200 μg); (**b**) Histology of liver and lung tissues of mice treated with ET or ET(H351A). Mice treated with EF in combination with PA died within 3 h, and were immediately necropsied. Mice treated EF(H351A) in combination with PA were euthanized 6 h later by CO_2_ inhalation and immediately necropsied. In the liver, sinusoid stenosis (**left** arrow), scattered necrotic lesions, and inflammatory cell infiltration (**right** arrow) were found in mice treated with EF(H351A) in combination with PA, while more marked necrotic lesions and inflammatory cell infiltration (**right** arrow) accompanied by hemorrhaging (**left** arrow) were identified in mice treated with EF in combination with and PA. In the lungs, hemorrhaging, edema, and inflammatory cell infiltration (arrows) were found around the trachea and bronchus in mice treated with EF in combination with PA but not in those treated with EF(H351A) in combination with PA; (**c**) and (**d**) cAMP levels in liver and lung tissues of mice treated with ET or ET (H351A). Mice were euthanized by CO_2_ inhalation 2 h after injection and immediately necropsied. Significant differences between the groups are indicated by *p*-values. The *p*-values of the indicated groups versus the solvent control group are shown. Data represent the mean ± standard error of the mean based on *n* = 3 mice per treatment. At least two biological replicates were performed for each experiment. (** *p* < 0.01, * *p* < 0.05).

In this study, we challenged each mouse with 50 μg of the wild-type or mutant EF in combination with 100 μg PA, and mice challenged with the individual proteins served as controls. The survival curves shown in Figure 4a show that EF(H351A) or EF(H351R) in combination with PA were not lethal and did not cause significant signs of illness, while 100% mortality was observed in mice within 12 h of challenge with an equal dose of wild-type EF combined with PA. When the doses of toxins was doubled (100 μg wild-type or mutant EF in combination with 200 μg PA), all animals in the EF and PA treated group died with 3 h of administration, while none died in the groups treated with EF(H351A) or EF(H351R) in combination with PA. However, histological analysis revealed some unexpected lesions induced by EF(H351A) and EF(H351R). The liver is considered to be the key target of ET-induced lethality and deletion of anthrax toxin receptor CMG2 from hepatocytes in mice led to remarkable resistance to ET [14]. In this study, necrotic areas in the liver were observed both in mice treated with the wild-type and mutant ET, while the lesions induced by EF(H351A) were relatively mild (Figure 4b). Furthermore, serious damage was found in the lungs of wild-type EF treated mice but not in those treated with the mutant toxins. cAMP levels within the liver and lung tissues in 2 h after the injection of the wild-type and mutant ET were also determined (Figure 4c,d). The cAMP amount in liver but not in lung was elevated by ET (H351A), while that in both of the organs were greatly increased by wild-type ET.

Together, these results clearly demonstrate that the mutated EF(H351A), which is generally used as a “loss” of AC activity control, retains limited AC activity and is associated with systemic toxicity, although the effects are significantly weaker than those of the wild-type EF. As reported in a previous study [13], mutants such as EF(H351A) which retain PA-binding ability represent potential toxin decoys, but our findings demonstrate that the liver toxicity of these mutants should be carefully considered for such applications. Furthermore, our results indicate that the mice footpad edema model is a highly sensitive method to determine the remaining AC activity of these mutants.

## 3. Experimental Section

### 3.1. Materials

All chemicals were reagent-grade or better and purchased from Sigma-Aldrich (Steinheim, Germany) unless otherwise stated. Restriction enzymes, ligase, Phusion polymerase and standards for agarose gel electrophoresis, GeneRuler DNA Ladder Mix were obtained from New England BioLabs (Ipswich, UK). The *Escherichia coli* strain M15 was purchased from Dingguo (Beijing, China).

### 3.2. Plasmid Construction

Plasmid pQE30-EF, constructed by insertion of the full-length wild-type EF gene with a six-histidine coding sequence at its 5′ end into vector pQE30, was used to express wild-type EF protein and as a template for generation of the single amino acid mutant plasmids. The codons encoding residue His351 in pQE30-EF were mutated to alanine (A) or arginine (R) by PCR using primer pairs 5-*Stu*I-EF and 3-*Apa*I-H351A or 5-*Stu*I-EF and 3-*Apa*I-H351R, respectively, which were designed to add *Stu*I and *Apa*I sites to the 5′ or 3′ ends of the PCR product, respectively. The sequences of the forward and reverse primers are as follows:

**5-*Stu*I-EF**: 5′-TTTAGGCCTGTTAATAAG-3′
**3-*Apa*I-H351A**: 5′-CAGGGCCCCAATCCGAACTCTTTCC**CGC**AACATTCAATC-3′
**3-*Apa*I-H351R**: 5′-CAGGGCCCCAATCCGAACTCTTTCCACGAACATTCAATC-3′

Mutant codons are highlighted by bold face and underlining indicates restriction enzyme recognition sites. The amplified PCR products were purified (High Pure PCR Product Purification Kit, Roche, Mammheim, Germany) and digested with the restriction enzymes *Stu*I and *Apa*I (NEB). After further purification, the digested enzymes were ligated to *Stu*I- and *Apa*I-digested plasmid pQE30-EF with T4 ligase. The newly constructed plasmids were designated pQE30-EF(H351A) and pQE30-EF(H351R) and verified by sequencing (Sangon, Shanghai, China). Each of the three plasmids included a His_6_-tag at the *N*-terminal of the gene, expressed under the control of a T5 promoter and tightly regulated by the *lac* operator.

### 3.3. Expression and Purification of EF and Mutant Proteins

Plasmids pQE30-EF, pQE30-EF(H351A) and pQE30-EF(H351R) were transformed into *E. coli* M15 competent cells to express EF and mutant proteins. Cells bearing the expression plasmids grown at 37 °C and 220 r/min in double-concentrated YT medium (16 g/L peptone from casein, 10 g/L yeast extract, 5 g/L NaCl), in the presence of ampicillin (100 μg/mL), were induced with 0.5 mM IPTG when A_600_ reached 0.6. Cells were harvested by centrifugation (4 °C, 30 min at 6000× *g*) after 4 h of induction. Protein expression was confirmed by analyzing crude lysates of induced samples in 10% SDS-PAGE. The *E. coli* biomass was resuspended in buffer A (100 mL buffer A per 1 L culture), followed by disruption in a French Press at 100 MPa. After centrifugation at 30,000× *g* for 30 min at 4 °C, the crude cell extract was separated from cell debris for protein purification. The hexa-histidine tagged wild-type and mutant proteins were separated using a Ni-NTA affinity column (5 mL His Trap HP) (GE Healthcare, Chicago, IL, USA) pre-equilibrated with buffer B1. The column was washed with buffer B2 after the supernatant was loaded and proteins were then eluted with buffer B3. The fractions containing the highest target protein purity indicated by SDS-PAGE analysis were pooled. After exchange of the medium to buffer C1 and concentration using an Amicon Ultra Centrifugal Filter (Millipore; Billerica, MA, USA). Device with a 30-kDa cut-off membrane, the target protein was further purified by SP-sepharose cation-exchange chromatography (GE Healthcare, Chicago, IL, USA) using a column pre-equilibrated with buffer C. The protein was eluted with the same buffer in a linear gradient of 0–250 mM NaCl. The constituents of buffers used in purification were listed in Table 1. Purified protein was obtained after high purity fractions were pooled and concentrated, followed by substitution of medium into phosphate-buffered saline (PBS). Endotoxin in the concentrated protein was removed with Detoxi-Gel™ Endotoxin Removing Gel (Thermo, Waltham, MA, USA) according to the recommended standard protocol. Protein concentrations were determined by BCA (Thermo, Waltham, MA, USA) assay using bovine serum albumin as the standard. Protein was stored in aliquots at −80 °C. The purity of a 10 μg sample of the concentrated protein was determined by SDS-PAGE analysis using the software Image J.

**Table 1:** Constituents of buffers used.

Solution	Ingredient	pH
Buffer A	50 mM sodium phosphate, 300 mM sodium chloride	8.0
Buffer B1	1% Glycerin and 2 mM β-mercaptoethanol added to buffer A	8.0
Buffer B2	20 mM imidazole added to buffer B1	8.0
Buffer B3	100 mM imidazole added to buffer B1	8.0
Buffer C	20 mM potassium phosphate, 1 mM EDTA, 1% glycerin and 2 mM β-mercaptoethanol	7.0

### 3.4. Intracellular cAMP Level Analysis

The activity of wild-type and mutant EFs(H351A and H351R) was measured by analysis of cAMP production in the CHO-K1 cell line. Cells were grown in minimum essential medium (MEM) supplemented with 10% fetal bovine serum, penicillin (100 U/mL), streptomycin (100 μg/mL), and glutamine (2 mM) at 37 °C under 5% CO_2_. Assays were carried out 24 h after cells were seeded in 96-well plates at a density of 2 × 10^5^ cells/well in the presence of various concentrations of recombinant proteins and PA (1 μg/mL). Following incubation for 1 h at 37 °C, total intracellular cAMP levels were assayed using the cAMP BioTrak enzyme immunoassay (EIA) System (GE Healthcare, Little Chalfont, UK) according to the manufacturer’s protocol.

### 3.5. Animal Experiments

Female C57BL/6 mice (aged 6–8 weeks) were purchased from the Laboratory Animal Centre in Beijing Institute of Biotechnology (Beijing, China). After a two-day period of acclimation, animals were used in experiments with access to food and water *ad libitum* and in appropriate environmental conditions. Mice were returned to their cages for recovery after any injection, and the morbid mice were euthanized by CO2 asphyxiation in euthanasia chambers or by cervical dislocation. All experiments were approved by the Animal Care and Use Committee of the Beijing Institute of Biotechnology (identification code: 20140017; date of approval: 20 May 2014).

### 3.6. Mouse Lethality Assay

For analysis of the potency of the wild-type and mutant EF, mice (*n* = 3 per group) were injected via the tail vein with 50 μg EF, EF(H351A), or EF(H351R) dissolved in sterile PBS to a final volume of 100 μL and combined with 100 μg PA or not. The survival of mice was monitored for 72 h.

### 3.7. Paw Edema Induction

For the footpad edema model, serial doses (0.5, 5, 50, 500, and 2500 ng) of EF, EF(H351A), and EF(H351R) combined with double amount of PA were diluted in PBS to a final volume of 25 μL and injected into the left hind footpad of the mice. The contralateral paw received the same volume of PBS. Other stimulators (forskolin, SQ22536, and H89) were diluted in DMSO to a concentration of 1 μg/μL. Paw edema was monitored before and after injection by measuring the diameter of the frontal area of the footpad with a caliper. The degree of edema was defined as follows:

edema degree = (*T_t_* − *T*_0_)/*T*_0_

where *T_t_* represents the thickness of the mouse foot at time-point t after injection, and *T*_0_ represents the thickness immediately prior to injection.

### 3.8. Histological Studies

Groups of mice were intravenously injected with 100 μg of EF, EF(H351A), or EF(H351R) combined with double amount of PA (*n* = 3) and, as controls, PBS (*n* = 3). The liver and lung were harvested were harvested, fixed in 10% formalin and embedded in paraffin. Light microscopy studies were performed on 3 mm tissue sections stained with hematoxylin and eosin (HE). Images were obtained with a E200 microscope (Nikon, Tokyo, Japan).

### 3.9. Tissue cAMP Level Analysis

For analysis of the cAMP level in the footpad, liver, and lung, the tissues were harvested and frozen in liquid nitrogen in 2 h after the mice were treated. Then the frozen tissues were ground to a fine power under liquid nitrogen. cAMP levels were assayed using the cAMP BioTrak enzyme immunoassay (EIA) System (GE Healthcare, Little Chalfont, UK) according to the manufacturer’s protocol.

### 3.10. Statistical Analysis

GraphPad Prism 6 was used for statistical analyses. Differences between the means of experimental groups were analyzed with the two-tailed Student’s *t*-test. *p*-values ≤ 0.05 were considered to indicate statistical significance.

## 4. Conclusions

In this study, wild-type EF and two mutants EF(H351A) and EF(H351R) were expressed and purified. Both of the mutants could mildly but not dramatically induce the cAMP in CHO cells, and were non-lethal to the experiment animals. But these variants of EF retained the ability to induce edema in the mice footpad model as well as cause lesions in the liver, accompanied with an elevation of cAMP level in the tissues. These results reflect the partial reduction in AC activity of the H351A and H351R mutants. In addition, the mouse footpad edema model is implicated as a sensitive and intuitive method for evaluating both ET toxicity and the efficacy of candidate therapeutic agents. An inhibition or relief of the ET induced footpad edema may indicate an effective treatment.
